# Cardiovascular imaging: a radiological perspective

**Published:** 2009-04

**Authors:** Bruce Spottiswoode, Jean Pierre De Villiers

**Affiliations:** MRC/UCT Medical Imaging Research Unit, Faculty of Health Sciences, University of Cape Town; Schnetler, Corbett and Partners Radiology, Bellville

Historically, conventional radiological imaging has had an important role in the diagnosis and imaging of acquired and congenital cardiovascular disease with modalities including chest radiography and fluoroscopy, and progressing to coronary catheterised angiography, echocardiography and nuclear medicine.^1^ Rapid technological advances and new clinical applications, coupled with an increase in therapeutic options for cardiovascular disease have resulted in a recent explosion in cardiovascular imaging.^2^

There is little doubt that multi-detector cardiac computed tomography (MDCT) and cardiac magnetic resonance imaging (MRI) are the hottest topics in cardiovascular imaging today. Although the number of 64-slice scanners and cardiac-enabled MRI units in clinical use is still relatively low, momentum is gathering fast as their versatility and striking capabilities become more apparent.

## MDCT

There is an ongoing evolution of ever faster and more sophisticated multi-detector CT technology and this has enabled the application of MDCT to a broader variety of clinical applications.^3^ The improvement of both spatial and temporal resolution of coronary CT angiography now approaches, and in some cases may have crossed, the threshold for routine imaging for atherosclerotic disease.^3^ As a rule of thumb, the number of acquired slices cannot be too thin and gantry rotation cannot be too fast. Gantry rotations of as low as 0.33 seconds are now the norm, as are sub-millimetre slice-acquisition thicknesses. New acquisition techniques have become standard and these include rate limitation, saline chasing, small field-of-view acquisition and dedicated acquisition filters.

Post-processing and evaluation of images have benefited from the availability of increased processing power and software advances made by the respective vendors. In addition to standard multi-planar reconstruction, axial imaging and three-dimensional volume-rendered images, most vendors now offer advanced visualisation tools that facilitate analysis of large-volume data sets. The object of most of these programs is to provide a means for rapid evaluation of the coronary vessels and rapid functional information, including ejection fraction. The programs provide automated extraction of the coronary arteries from the data set, allow unravelling of tortuous vessels and offer tools for quantitative evaluation of the severity of stenosis.

Sixty-four detector-row technology now appears to be the norm, as these units can provide isotropic resolution and have reduced breath-holds for acquisition to less than 20 seconds. They are, however, still not able to provide coverage of the heart in a single rotation. Dual-source or 320-slice units seem to be the next wave in technological advancement, and it will not be long before whole-organ evaluation and perfusion can be measured in a single rotation within a short breath-hold. These technologies will also allow more robustness in the imaging of patients with arrhythmias and rapid heart beats. With prospective triggering, a significant reduction in radiation dose has also become possible.

MDCT can be used to assess both cardiac structure and function, as well as evaluate disease in the field of view, outside of the heart and pericardium.^1^ Applications are, contrary to common belief, not limited to the evaluation of coronary artery stenosis. Additional applications include detection of vascular anomalies, evaluation of aneurysms, coronary vein mapping, characterisation of chamber morphology and function, imaging native and prosthetic valves and congenital heart disease, diagnosis of cardiac masses and pericardial disease, as well as assessment of post-operative complications.^1^,^2^,^4^,^5^ The appropriateness of these indications is comprehensibly addressed in a report published in the *Journal of the American College of Cardiology* in 2006 by Hendel *et al.*^1^

There are ongoing trials evaluating the use of MDCT to assess the response of coronary arterial plaque to high-dose statins, and this may yet prove to be a significant indication. Concerning evaluation of coronary vascular disease, the established and accepted role currently is in evaluation of intermediate-risk patients with chest pain where the negative predictive value in numerous studies approaches 100%.^5^

A number of challenges will determine the success and future use of the imaging modality, including issues relating to training of operators and radiation dose used. The American College of Radiologists recently published guidelines outlining recommendations relating to accreditation and safety.^1^ There are specific recommendations regarding training for radiologists and technicians and these include minimum requirements for training and cases reported. Specific guidelines exist for reducing radiation dose and these include automated X-ray dose-shaping algorithms and X-ray tube pulsing. The local fraternity of cardiologists and diagnostic radiologists would benefit from taking note of these guidelines as well as considering accreditation of local units.

## CMR

Cardiac magnetic resonance (CMR) imaging has evolved at a formidable pace over the past decade, and the modality now boasts a variety of morphological and functional applications. MRI is renowned for its ability to visualise soft tissue, a characteristic enhanced by the variety of different image-weighting techniques. There are, however, a plethora of other tricks and techniques that are available for a routine examination.

Most standard full-body MRI scanners can be upgraded for cardiac imaging with the addition of a chest radio-frequency coil and software comprising a series of cardiac pulse sequences and possibly post-processing tools. The radio-frequency chest coil serves to maximise the MRI signal. Parallel imaging is a relatively recent advent that makes use of multiple phased-array cardiac coils to significantly reduce the scan time with only a marginal trade-off in signal.^6^

With the advent of mainstream 3-Tesla MRI units, much research is being done to quantify the effectiveness of cardiac imaging at high field strengths. Signal-to-noise ratio (SNR) is effectively proportional to field strength, thus permitting higher spatial resolution and/or shorter scan times. There is unfortunately a trade-off in that several types of image artefacts become more pronounced at higher field strengths, and radio-frequency absorption limits place more stringent constraints on MRI sequence development at high fields. Kelle and Nagel^7^ provide a more detailed discussion of the advantages and limitations of cardiac MRI at 3 Tesla.

Black-blood imaging can be used to provide high-resolution structural images with clearly defined endocardial borders. Single-slice ciné images can be acquired in a short breath-hold and provide detailed information about wall motion. A short-axis stack of ciné images is typically acquired and post-processing is done to estimate ejection fraction, wall thickening, myocardial mass, and other related parameters. Controlled doses of dobutamine can be administered to investigate wall motion abnormalities in stress conditions. Gradient-echo ciné images are also useful for valvular imaging and have a niche application for measuring flow regurgitation, as slow-moving or turbulent blood is clearly visible.

MRI provides excellent tissue contrast information and therefore is key to diagnosing certain cardiomyopathies, including arrhythmogenic right ventricular cardiomyopathy (ARVC). MRI is also useful for distinguishing between constrictive pericarditis and restrictive cardiomyopathy. Iron deposits associated with, for example thalassaemia, can be clearly imaged using T2* mapping.

MRI viability imaging is unparalleled by the other imaging modalities. Gadolinium contrast agents settle in oedematous areas and also take longer to be washed out of scar tissue. Delayed-enhancement MRI involves imaging the heart 10 minutes after administering the contrast. Infarcted tissue is bright in these images and both the extent and severity of the scar tissue are easily identifiable. Low-dose dobutamine stress tests in MRI also add useful insight in distinguishing between viable and non-viable tissue. A more striking way of portraying delayed enhancement is using inversion recovery sequences that null myocardial tissue and thus enhance the contrast of the damaged tissue.

Myocardial perfusion can be quantified by rapidly measuring the uptake of gadolinium. A ciné image series with manually introduced contours is processed to yield regional measures of tissue perfusion. In contrast to the longer-term gadolinium uptake, ischaemic myocardium appears darker than normal myocardium in the first images, due to reduced perfusion. A more recent and accurate perfusion measurement technique involves a dual bolus, whereby a large bolus of gadolinium is administered a short time after a small bolus.^8^

Phase-contract (PC) velocity encoding provides an instantaneous measure of velocity. This can be used to provide flow–time curves through the valves and the great vessels.^9^ The technique can also be applied to the myocardium, yielding regional measures of velocity and strain rate.^10^,^11^

In MRI tagging, the myocardium is modulated by a series of saturated dark bands or tags. These are a material property of the tissue and can be seen to deform as the heart moves. Tagged images provide insight into contractility and myocardial mechanics, and can be processed to yield meaningful measures of myocardial strain.^7^ Displacement encoding with stimulated echoes (DENSE) is a more recent technique that provides more accurate measures of displacement and hence myocardial strain.^6^,^12^ Both myocardial tagging and DENSE allow a user to track discrete portions of tissue as they traverse the cardiac cycle.

Myocardial dyssynchrony can be mapped by using tagging or DENSE regional strain–time curves. Dyssynchronous but healthy segments of myocardium can be measured using a variety of techniques, such as the delay to onset of strain contraction. This technique can be used in combination with delayed-contrast MRI to improve the planning of cardiac resynchronisation therapy.

CMR has already gained recognition as a powerful and versatile modality, and the battery of potentially useful applications continues to grow. The following techniques are still in their infancy, but give a good idea of the future potential of this evolving field.

Myocardial fibre structure can be quantified using diffusion-tensor imaging (DTI), which is an extension of diffusionweighted imaging.^13^ Although lengthy scan times currently limit the technique for *in vivo* use, it has potential for identifying disease-associated disruptions in myofibre orientation. Measures of myocardial elasticity (or stiffness) can be obtained using MR elastography,^14^ which makes use of a carefully controlled externally applied mechanical excitation.

Perfusion can be measured without contrast agents, using arterial spin labelling (ASL), which magnetically labels the blood flowing into an imaged slice. The technique is non-invasive but has a lower sensitivity than the gadolinium-based methods.

MR cardiac spectroscopy^15^ is capable of quantifying regional metabolic function by measuring compounds such as phosphates and lipids, which are present in metabolic pathways. For example, ^31^P spectroscopy has an anticipated future in viability imaging where ischaemic myocardium will portray decreased energy metabolism.^16^

Sodium MR imaging^17^ demonstrates signal enhancement in infarcted tissue as a result of increased intracellular sodium occurring with myocyte cell membrane breakdown.

Interventional MRI been constrained to a few research sites and has been limited by the small bore of commonly available MRI units, and stringent limitations on the use of ferrous materials. A selection of new wide-bore MRI scanners has recently been released and these alleviate some of the restrictions and support this growing avenue.

## Conclusion

The current MDCT and cardiac MRI techniques and technological advances have revolutionised the approach to imaging of cardiovascular disease. The techniques have proven themselves to be robust and safe, providing existing international guidelines are followed.

The quantum advances in these imaging modalities have allowed radiologists to once again contribute to the imaging of cardiovascular disease but have, and will continue to contribute to turf battles regarding ownership of the modalities. At present, the old adage ‘use it or lose it’ would seem to be appropriate.
